# A critical evaluation of the electronic surgical logbook

**DOI:** 10.1186/1472-6920-6-15

**Published:** 2006-03-01

**Authors:** R Achuthan, K Grover, J MacFie

**Affiliations:** 1 Department of General Surgery, Scarborough General Hospital, Scarborough Yorkshire, YO12 6QL, UK

## Abstract

**Background:**

The Association of Surgeons of Great Britain and Ireland (ASGBI) devised the electronic surgical logbook (version 2.4) for higher trainees in General Surgery enabling trainees to compile a uniform data set of their operative and training experience. This is in use by higher surgical trainees (HST) in the United Kingdom. This logbook permits trainees to submit data centrally into a Regional Analysis Database (RAD). With the implementation of the European Working Time Directive (EWTD) there is need for reliable data to assess the effects of the directive on training. In order to draw meaningful conclusions from the database the quality of data needs to be validated. We critically analysed the RAD in the Yorkshire region for a one-year period.

**Methods:**

The RAD from the ASGBI for the Yorkshire region was analysed. Data for the period 01/10/2002–30/09/2003 was identified and interrogated using Microsoft Excel (2000 version). The RAD was compared with information obtained from the Regional Surgical Advisor for Yorkshire with respect to hospitals, surgical consultants and HST's in the region during the study period.

**Results:**

There were 13,755 operations entered for the study period. 579 corrections to the data had to be made (4.2%) and a further 1140 entries were deleted (8.2%). Following corrections and deletions 12,615 operative entries were available for analysis. Overall 12.5% of the data required either correction or exclusion from the database prior to analysis.

**Conclusion:**

The RAD has a large dataset useful to monitor and assess training. However, the quality of the data needs to be verified prior to use. Recommendations have been made to develop the ASGBI logbook, which would eventually translate to improved data reliability of the RAD.

## Background

The electronic surgeon's logbook for Higher Trainees in General Surgery was initially devised by the Specialist Advisory Committee in General Surgery (SAC) and the Association of Surgeons in Training (ASiT)[1]. This did away with paper based logbooks and brought about some uniformity in collection of data. The Association of Surgeons of Great Britain and Ireland (ASGBI) have further developed it and the surgical trainees in the UK currently use this logbook.

The Surgeon's Logbook Database permits the trainee to record operations performed, procedures performed and courses completed. This provides a comprehensive dataset, which can potentially provide useful information regarding the level of operative training and supervision provided to the surgical trainee. Trainees can obtain copies of the programmes from their regional training advisor or from the ASGBI web site [2].

The logbook allows trainees to submit a standard data set of their operative and training experience centrally to the SAC. The eventual aim of the central database (The Regional Analysis Database) being the assessment of a trainee's progress and assessment of the quality of training provided. Similar computer-based logbooks have been developed in Australia and have been found to be a convenient and versatile method of record keeping [3].

The Regional Analysis Database is designed for use by regional training co-ordinators to analyse the data collected by the trainees in a given region. It uses Microsoft Access version 97 for analysis of the data. This database relies on the trainees of a given region to submit their logbook data centrally at regular intervals. The regional analysis database at present has contains 300,000 operative episodes entered over a 5-year period [1]. This is a huge dataset and would indeed be a powerful tool in assessment of surgical training provided in the UK. However it would be unwise to make any conclusions from the database without first attempting to validate the quality of the data.

A literature review on Medline did not identify any information regarding the ASGBI Logbook Regional Analysis Database. Evidently no validation of this database has been performed to date.

The aim of this study was to analyse and validate the data obtained from The Regional Analysis Database from the Association of Surgeons of Great Britain and Ireland for the Yorkshire region.

## Methods

The Regional Analysis Database from the Association of Surgeons of Great Britain and Ireland for the Yorkshire region was analysed for the period 01/10/2002 – 30/09/2003. The data for the study period was interrogated using Microsoft Excel (2000 version).

Data from the Regional Analysis Database was scrutinised for three variables that could be readily cross checked i.e. hospital, consultant and trainee details. This data was then corroborated with information available from the Regional Advisor for Yorkshire with regards to the details of the surgical trainees, hospitals included in the HST rotation programme and also for the substantive consultants in these hospitals who were involved with the training of the surgical registrars.

## Results

**Figure 1:**
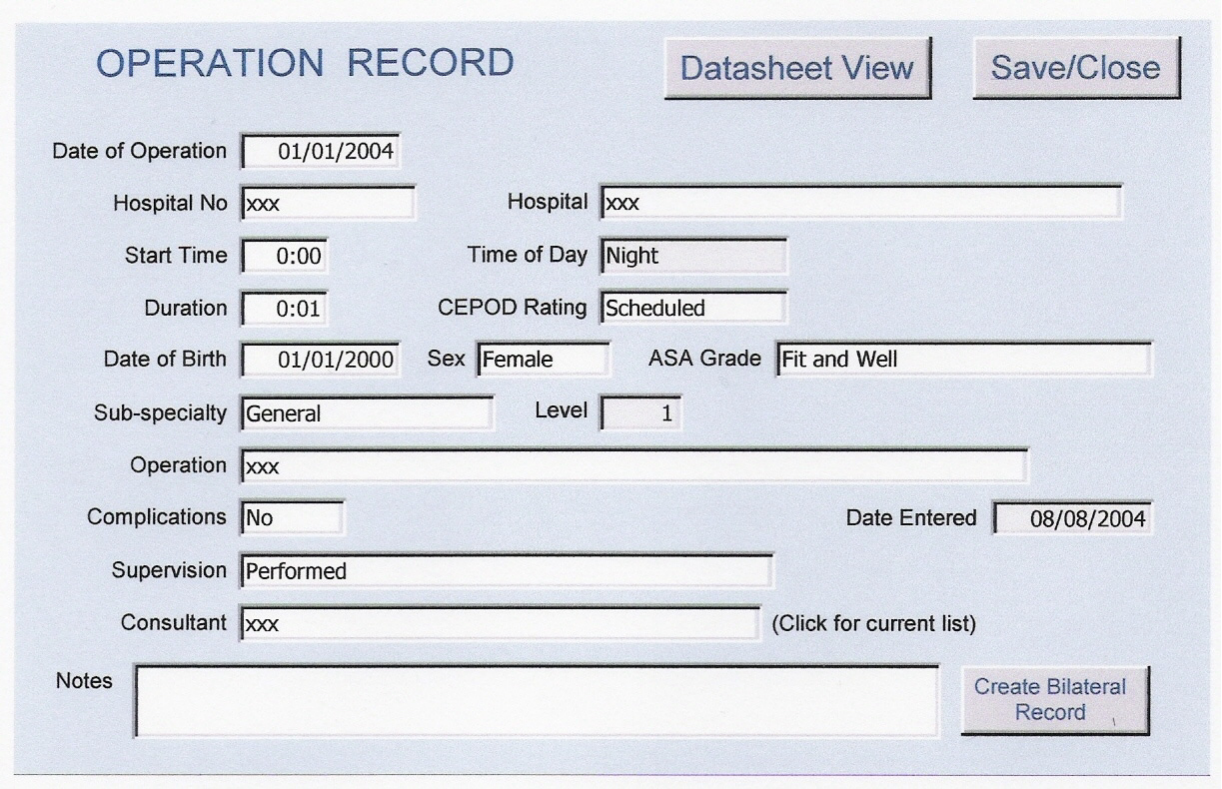
Microsoft Access version of the ASGBI logbook (version 2.4). The various fields for collecting the data are demonstrated.

An analysis was performed for the operative details held in the Regional Analysis Database for Yorkshire during the 1-year study period. A total of 13,755 operations were identified. The details provided by the database for each procedure included the date of surgery, hospital, patient details including hospital number, date of birth, sex, ASA grade (with the following fields: 1-Fit and well, 2-Mild systemic disease, 3-Signifcant systemic disease, 4-Life threatening disease and 5-Not expected to survive 24 hrs), CEPOD classification of the operation (with the following fields: scheduled, urgent and emergency), start time and duration of surgery, time of day (with the following fields: Day, Evening and Night), speciality concerned (with the following fields: Breast, Endocrine, General, Coloproctology, Laparoscopic, UpperGI/Hepatobilliary, Paediatrics, Emergency, Transplant and Vascular) details of the surgical procedure, level of complexity of the procedure (with the following fields: 1, 2 and 3) consultant involved, the level of supervision (with the following fields: Assisting, Performed, Supervised with trainer scrubbed, Supervised with trainer unscrubbed but in theatre and Training juniors) and complications (with the following fields: Yes and No) (figure 1).

In this analysis the variables validated were the hospital details, the consultant details and the trainees submitting the data. This was because these details were also available from the Regional Surgical Advisor and could be therefore be readily cross-checked. The other parameters were dependent on the trainee for the quality of input and reliability and there was no means to crosscheck these fields.

### Hospitals

There are currently 17 hospitals in Yorkshire that have surgical trainees as part of the HST rotation. The database had data pertaining to 16 of these with no data from 1 hospital. On interrogating the data further, hospital names were found to lack uniformity. Firstly, there was a lack of consistency in the naming of hospitals by the trainees e.g. Bradford Royal Infirmary appeared as Bradford or Bradford Royal. Secondly, there was lack of uniformity when it came to peripheral hospitals attached to training hospitals, with some trainees entering procedures performed at those sites separately e.g. entries were present for Chapel Allerton Hospital attached to Leeds General Infirmary but no entries were found for St. Luke's Hospital attached with Bradford Royal Infirmary. Thirdly, private hospitals had been included, though only on 2 occasions. Lastly, hospitals from outside the region were included. Overall 409 corrections were required to make the hospital names accurate. 319 entries were deleted as they involved hospitals outside the region.

### Consultants

On analysis of the consultants involved in the training programme problems were identified with entry of wrong consultants for a given hospital. This is probably secondary to input error. Another input error was the different ways the name of the same consultant was entered by various trainees, e.g. Professor S. Homer-Vanniasinkam had entries as Homer-Vanniasinkam S and Homer S. This meant that when the data was analysed each of these versions was counted as a different individual. 170 corrections were made to rectify these errors and also to correct entries for consultants who worked across 2 sites e.g. St James's Hospital and Leeds General Infirmary. This obviously has implications for the trainer if the training workload is assessed without correcting for this factor.

A major difficulty encountered was entry of locum consultants by name rather than as "locum consultant". This would impact quite significantly as the training provided by "locum consultants" would be hugely underestimated if the database was interrogated without correction. There were only 82 entries from the 13,755 that specifically had used "locum consultants" as a field rather than naming the consultant. There were 469 entries, which required deletion, as the training consultant did not match the substantive list from the Regional Advisor. These presumably would be "locum consultants" but as there was uncertainty these were excluded to ensure that the dataset was as clean as possible. A further 113 entries were deleted as they involved consultants from different specialities who had operated out of hours for emergencies i.e. cardio thoracic consultant called in for chest injury, vascular cross site cover by a consultant from a different hospital.

### Trainees

Two trainees had not submitted their operative logbook. Coincidentally, they were rotating at the same hospital resulting in that hospital, not being represented in the Regional Analysis Database. Two other trainees who were out of programme (on study leave for a 1-year period)) had provided information, as had 3 trainees who were not on the list of The Higher Surgical Trainees for the region obtained from the Regional Advisor. Another problem was submission of operative experience from research posts by newly appointed trainees. Overall a total of 239 deletions were needed to amend these entries.

Thus a final figure of 12,615 operations remained after correcting the database for hospitals, consultants and trainees.

### Logbook fields

During the analysis it was noted that certain fields in the database were modified. One such field was "operative procedure". The logbook (version 2.4) has 367 operations listed. However, the regional analysis database had 457 operations. Addition of fields to any database has adverse effects when it comes to analysis of data.

## Discussion

The data recorded in log books has two important uses, firstly it provides a record of training received by a trainee and secondly pooled logbook data can provide a large data set to monitor and assess training.

Numerous paper-based logbooks have been around for years and have provided a good record of individual experience. Some like the European Board of Surgery Qualification in Vascular Surgery [4] have a requirement for a fixed number of indicator procedures with credit points for each, that have to be performed before completion of training can be certified. However the main draw back of these was the difficulty in centralising the information. The ASGBI and other institutions involved in developing the electronic surgical logbook deserve credit in that mechanisms were put in place to collect data centrally.

The Regional Analysis Database is a source of a very large case-mix of operative experiences of the surgical trainees in the UK. With about 300,000 operative episodes this could provide very interesting and useful data regarding the level and adequacy of surgical training provided. There is great need for reliable data to assess the impact of the European Working Time Directive on training. In order to validate the quality of data the Regional Analysis Database information was compared with data obtained from the Regional Advisor for Yorkshire. This was done as the Regional Advisor has the most current and accurate information pertaining to the training hospitals, training consultants and the higher surgical trainees.

The Regional Analysis Database had a total of 13,755 operative procedures for the period between 01/10/2002 and 30/09/2003.

Analysis of the information base for the regional hospitals identified that data had been obtained from 16 of the 17 training hospitals in the region. A lack of uniformity was evident in the entry details for hospital names. Corrections were required in 409 entries. 319 entries were deleted as they involved hospitals outside the region and private hospitals. A total of 13,436 operations remained after compensating for hospital details.

Analysis of the consultant details revealed inaccuracies. A total of 170 corrections were required. A further 582 entries were deleted as they involved consultants not on the list of substantive consultants involved in the training programme for the region. Following these corrections 12,854 entries remained.

Inaccuracies in trainee data led to a total of 239 entries being deleted. Overall 1140 entries were deleted and 579 corrections were made. Thus a final figure of 12,615 operations remained after correcting the database for hospitals, consultants and trainees. In this analysis the data from the other fields in the logbook were not scrutinised, as there is no simple comparator to validate these with. It is however concerning that 12.5% of the data needed corrections for simple fields such as hospital name, consultant details and trainee details.

### Deficiencies and recommendations

We find the inaccuracies in the database were primarily due to the design of the logbook, which permits the trainee to add and remove fields in the various data collecting categories. For the data to be robust the number of fields allowing free text needs to be minimised. This problem is not unique to the surgical logbook but also exists in those of other specialities. The logbook prepared by the Royal College of Anaesthetists suffers from a similar malady [5].

A solution would be to have a default list of hospitals and consultants for a region thereby ensuring uniformity. Similarly the list of operative procedures should also be set as a default list not permitting any additions. Logbooks used in some other countries e.g. in Australia, have recognised that default lists, which do not permit addition of categories by the trainees, minimises mis-categorisation and transcription errors [3]. It would be important to have a robust mechanism to regularly check and update operations and names of consultants to the logbook default lists so as to prevent trainees from making additions.

Some of these deficiencies have been addressed in the newer V3 logbook, which does not allow additions to operative procedures and has a default list of hospitals and consultants for a region. The new version however, does allow for addition and changes to be made for hospital names and consultant names and additionally names the locum consultants rather than having a separate category for "locum consultants". The web-based version of the logbook has gone some way in trying to address these deficiencies.

Data entry in the logbook should be kept as simple as possible. It would be helpful to attempt and minimise fields which may not be relevant but can lead to inaccurate entries e.g. ASA category, sex of patient, start time of operation, date of birth of patient, hospital number of patient. These fields do not add to the final analysis in terms of training and if collected inaccurately would be eventually meaningless.

Another source of input error is the trainee i.e. trainees submitting data of their experience prior to obtaining a training number, out of programme experience, non compliance in data submission, inaccurate compilation of data etc. This can however potentially be overcome by providing verbal and written advice to the trainee prior to submitting individual logbook data centrally and by regular checks of operative experience at appraisals and in RITA's (record of in training assessments).

### Key recommendations

• Minimize free text

• Default lists for hospitals, consultants & operations

• Introduce field 'locum consultant'

• Simplify data entry

• Clear instructions to trainees regarding data entry

## Conclusion

The Regional analysis database is a source of very valuable information. This exercise however, raised concerns about the quality of the data, with 12.5% of the data entered needing to be corrected before being available for analysis. Conclusions drawn on the basis of poor quality data can be erroneous. With improvements to the logbook and implementation of some of the recommendations, the quality of data can be improved which will allow making meaningful recommendations on training issues. Only with validated good quality data can the Regional Analysis Database fulfil its aspirational role of being a good tool to monitor and regulate training.

## Competing interests

The authors confirm that there are no financial or other conflicts of interest in the writing of this paper.

The findings of this study were presented at the Annual Meeting of The Association of Surgeons of Great Britain and Ireland held in Glasgow in April 2005.

## Authors' contributions

All authors read and approved the final manuscript.

RA was involved in the study design, analysis of data and drafting of the manuscript.

KG was involved in the study design, analysis of data and drafting of the manuscript.

JM was involved in the study concept and design, critical review of the manuscript and is the corresponding author.

## Pre-publication history

The pre-publication history for this paper can be accessed here:
